# Anti-glycation, Carbonyl Trapping and Anti-inflammatory Activities of Chrysin Derivatives

**DOI:** 10.3390/molecules23071752

**Published:** 2018-07-17

**Authors:** Seung Hwan Hwang, Hyun Yong Kim, Guanglei Zuo, Zhiqiang Wang, Jae-Yong Lee, Soon Sung Lim

**Affiliations:** 1 Department of Food Science and Nutrition, Hallym University, 1 Hallymdeahak-gil, Chuncheon 24252, Korea; isohsh@gmail.com (S.H.H.); khy9514@nate.com (H.Y.K.); guangleizuo@foxmail.com (G.Z.); Wang290123@aliyun.com (Z.W.); 2 College of Public Health, Hebei University, Baoding 071002, China; 3 Department of Biochemistry, School of Medicine, Hallym University, 1 Hallymdeahak-gil, Chuncheon 24252, Korea; jyolee@hallym.ac.kr

**Keywords:** chrysin, advanced glycation end products, methylglyoxal trapping, 5,7-di-*O*-acetylchrysin, inflammation

## Abstract

The aim of this study was searching anti-glycation, carbonyl trapping and anti-inflammatory activities of chrysin derivatives. The inhibitory effect of chrysin on advanced glycation end-products (AGEs) was investigated by trapping methylglyoxal (MGO), and MGO-conjugated adducts of chrysin were analyzed using LC-MS/MS. The mono- or di-MGO-conjugated adducts of chrysin were present at 63.86 and 29.69% upon 48 h of incubation at a chrysin:MGO ratio of 1:10. The MGO adducted positions on chrysin were at carbon 6 or 6 & 8 in the A ring by classic aldol condensation. To provide applicable knowledge for developing chrysin derivatives as AGE inhibitors, we synthesized several *O*-alkyl or ester derivatives of chrysin and compared their AGE formation inhibitory, anti-inflammatory, and water solubility characteristics. The results showed that 5,7-di-*O*-acetylchrysin possessed higher AGE inhibitory and water solubility qualities than original chrysin, and retained the anti-inflammation activity. These results suggested that 5,7-di-*O*-acetylchrysin could be a potent functional food ingredient as an AGE inhibitor and anti-inflammatory agent, and promotes the development of the use of chrysin in functional foods.

## 1. Introduction

Protein glycation (PG) is a non-enzymatic reaction that occurs initially from several reactions between a reducing sugar and a free amino group, representing one of the main pathways related to the development and progression of various diabetic complications such as nephropathy, retinopathy, and neuropathy [1]. Advanced glycation end products (AGEs) are irreversibly produced from the glycation process. The resulting products are unstable and can react with other free amino groups, causing protein modifications including alternative protein half-life and altered immune system and enzyme functions, leading to pathophysiological changes [2]. Intracellular AGEs play important roles as stimuli for activating intracellular signaling pathways, as well as for modifying intracellular protein function, altering receptor recognition, and generating oxidative stress and carbonyl stress [3]. Many studies have revealed the vital role of PG in the pathogenesis of age-related diseases such as diabetes, atherosclerosis, end-stage renal disease, and neurodegenerative disease from synthetic and natural sources [4].

Flavonoids are polyphenolic compounds that are categorized into flavonols, flavones, flavanones, isoflavones, catechins, anthocyanidins, and chalcones. Flavonoids have gained considerable attention due to their anti-glycation physiological functions [5]. Various polyphenols like quercetin, genestein, tannic acid, and gallic acid showed inhibition of glycoxidation [6], and baicalin exposed antioxidant activity as flavonoid [7], glycosylated flavonoids showed more resistant effect than the aglycon form at heat treatment [8].

Chrysin (5,7-dihydroxyflavone, CS), a naturally occurring flavone, has multiple biological activities related to diabetes, affecting diabetic renal tubulointerstitial fibrosis, inhibiting glomerulosclerosis, and anti-inflammatory activity [9,10,11,12,13].

Among the many reactive dicarbonyl compounds and AGE precursors, methylglyoxal (MGO) is a significant contributor to intracellular AGE formation because of its high reactivity and origin diversity in in vivo conditions [11,14]. MGO can easily react with cysteine and lysine residues of proteins, resulting in protein modification and DNA damage, and eventual, cytotoxicity and oxidative stress [15]. One of the working mechanisms of inhibition of AGEs by flavonoids is that flavonoids can block the formation of Schiff bases that trap reactive carbonyl intermediates (middle stage) and block the formation of Schiff bases (early stage) or AGEs (last stage) [16].

Many studies have revealed that flavonoids can efficiently inhibit DNA glycation and suppress reactive carbonyl compound-induced PG [16,17]. However, the mechanism underlying the anti-glycation effect of CS is still largely unknown, and no data have yet been published on the inhibitory effects of CS and its derivatives on AGEs. Therefore, we investigated CS inhibitory activity on AGE formation, analyzed the MGO-adducted structure including a kinetic study of the trapping of MGO adducts, and confirmed the attachment of MGO-conjugated adduct position at CS by liquid chromatography mass spectrometry (LC-MS/MS) and nuclear magnetic resonance (NMR). Additionally, we synthesized several *O*-alkyl or ester derivatives of CS to develop potential AGE inhibitors and evaluated the feasibility of these beneficial materials as functional food sources regarding AGEs formation, anti-inflammation, and water solubility.

## 2. Results

### 2.1. Inhibitory Effects of Chrysin on Amadori Compound and AGE Formation

**Figure 1:**
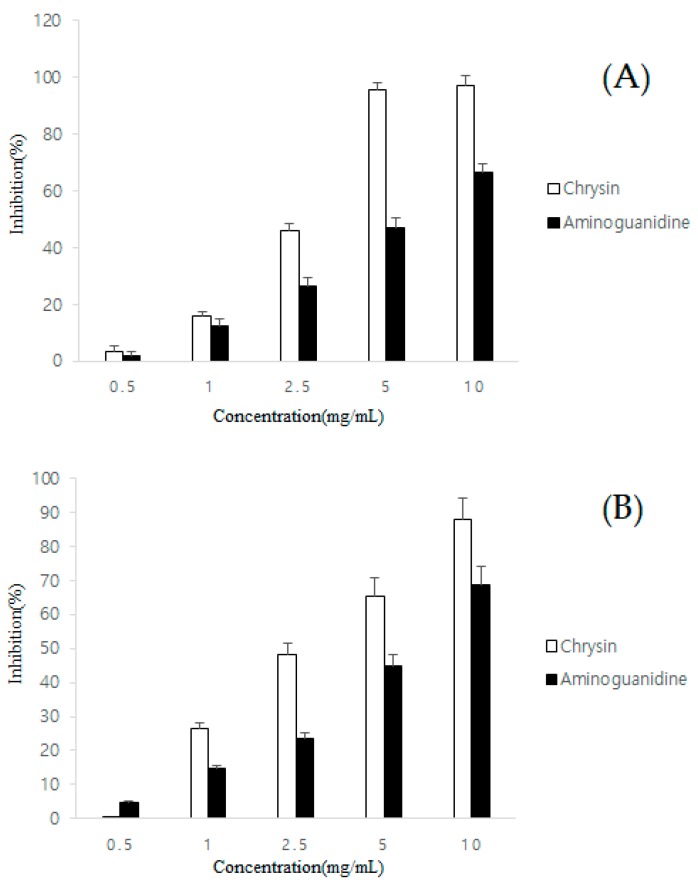
Inhibitory effect of various chrysin concentrations on amadori compound (**A**) and AGEs formation (**B**). Aminoguanidine is the positive control for AGEs.

The CS was preliminarily evaluated by examining Amadori compound and AGE formation, and CS inhibited the formation of both these chemical species. CS displayed dose-dependent inhibition rates at different concentrations, on Amadori compound formation, and AGE formation. The IC_50_ values of CS were 17.41 and 24.96 μM (Figure 1A,B) for Amadori compound and AGE formation, respectively.

### 2.2. Identification of the Chrysin MGO-conjugated Adducts by LC-MS/MS

The CS MGO-conjugated adducts were analyzed from an incubated mixture (48 h) of CS and MGO at a ratio of 1:10. After incubating, two high polarity peaks were detected at 25.11 and 25.70 min (CS is 31.56 min, data not shown). The 25.11 min peak exhibited molecular ion *m*/*z* values of 327 [M + H]^+^ and 349 [M + Na]^+^, which are 72 and 95 mass units higher than that of CS (*m*/*z* 254). In addition, the 25.11 min peak had a fragment ion *m*/*z* 309 [M + H-H_2_O]^+^, suggesting it lost one H_2_O (*m*/*z* 18) molecule. Another fragment was observed at ion *m*/*z* of 279 [M + H-2H_2_O-12]^+^. Based on the data, we determined that the 25.11 min peak was the mono-MGO adduct of CS. Additionally, the 25.70 min peak displayed molecular ion *m*/*z* values of 399 [M + H]^+^ and 421 [M + Na]^+^, which are 144 and 167 mass units higher, respectively, than those of CS. This peak had molecular ion *m*/*z* values of 381 [M + H-H_2_O]^+^, 363 [M + H-2H_2_O]^+^, and 303 [M + H-2H_2_O-24]^+^, suggesting this compound was a di-MGO-conjugated CS.

### 2.3. Structural Elucidation of the Chrysin Mono- and di-MGO Adducts by NMR

Positions of the CS MGO-conjugated adducts were not confirmed by LC-MS. Therefore, the CS MGO-conjugated adducts were subjected to recycle HPLC with H_2_O-MeOH (0–25%) as the eluent to give mono- and di-MGO from the incubation mixture (48 h) of CS and MGO at a ratio of 1:10. We analyzed the molecular structure of purified MGO-conjugated adducts using ^1^H and ^13^C-NMR including HMBC. The ^1^H-NMR spectrum of the –mono MGO adduct showed two singlet signals for two protons instead of the three proton signals which were observed in the ^1^H-NMR spectrum of -mono MGO adduct with signals of the MGO group, suggesting that MGO-conjugated with CS at position 8 of the A ring. The ^13^C and HMBC spectra were utilized to identify the position of the -mono MGO adduct; a long-range correlation between H-11/C-8 confirmed the attachment of the -mono MGO adduct at C-8 (97.3 ppm). Other useful correlations between H-11/C-7, 9, 12, 13, H-13/C-12, and H-6/C-5, 7 confirmed the position of the attachment (Table 1).

The ^1^H and ^13^C-NMR data confirmed the di-MGO adduct. The data indicated the presence of three proton signals in CS; however, only a single signal proton signal at δH 6.86 (1H, s, H-3) ppm in di-MGO adduct was observed. Likewise, di-MGO adduct proton signals did not affect the five proton of C ring, suggesting that di-MGO-conjugated with CS at position 8 and 6 of the A ring. The di-MGO adducts were assigned the long-range correlation between at δH5.18 (1H, s, H-11)-δC 103.7 (C-8) ppm and δH 5.17 (1H, s, H-14)-δC 103.2 (C-6) ppm in the 8 and 6 positions in A ring by HMBC, respectively. Additionally, a long-range correlation between H-11/C-7, 9, 12, H-13, 16/C-12, and H-14/C-5, 7, 15, 16 reconfirmed the attachment at positions 8 and 6 (Table 1).

### 2.4. Kinetic Study on the Trapping of the MGO-conjugated Adducts on the Formation of AGEs by Chrysin

Next, we conducted a kinetic study on the trapping of the CS MGO-conjugated adducts using HPLC after incubation of CS with MGO at five different ratios and times. The CS–mono MGO adduct displayed high adduct formation, with ratios of 8% at 1:0.1, 21% at 1:0.5, 30% at 1:1, and 36% at 1:5 within 12 h (Table 2A).

The CS–di MGO adduct increased in a low dose-dependent manner between 0.2–3% with ratios shown in Table 2B. However, the mono-MGO and di-MGO adducts were trapped high 42 and 6% within 12 h in 10 mM MGO, and the trapping efficiency increased to 64 and 30%, respectively, when incubated for 48 h. CS appeared to trap MGO much more efficiently than low MGO concentration when high MGO concentrations occurred in the same system.

### 2.5. Inhibitory Effect of Chrysin Derivatives on Each AGE Formation Stage

#### 2.5.1. Amadori Compound Formation

We compared the abilities of CS and its derivatives to inhibit Amadori compound formation. Among the six compounds, CS showed the strongest inhibitory activity on Amadori compound formation, which was 6.30-fold higher than that of Aminoguanidine hydrochloride (AG) (IC_50_ = 109.74 μM). In addition, the inhibitory activity of 7-*O*-A and 5,7-*O*-DM on early stage was 4.66- and 13.92-fold higher than that of the positive control (Table 3).

The 5,7-*O*-DA displayed the strongest inhibitory activity on Amadori compound formation, which was 66.96 and 422.07-fold higher than that CS and positive control. 5,7-*O*-DA displayed dose-dependent inhibition rates of 1.76–55.13% at 0.0025–0.05 μg/mL. On the other hand, 7-*O*-M and 7-*O*-P exhibited no inhibitory activities up to a concentration of 10.0 mg/mL.

#### 2.5.2. AGE Formation

The inhibitory activities of CS and derivatives were assayed against AGE formation, and the results are presented in Table 3. The known AGE inhibitor AG (IC_50_ = 136.79 μM) was used as a positive control; CS exhibited 5.48-fold higher activity than that of AG. Among the compounds examined, 7-*O*-A, 5,7-*O*-DA, and 5,7-*O*-DM exhibited strong activity with IC_50_ values of 21.61, 0.91, and 41.78 μM, respectively. Especially, 5,7-*O*-DA showed inhibitory activity at low concentrations (Table 3). In the same manner, we examined the effects of 7-*O*-M and 7-*O*-P on AGE formation, however 7-*O*-M and 7-*O*-P showed low inhibitory activity on AGE formation (i.e., their IC_50_ values exceeded 200 μM).

#### 2.5.3. AGEs Cross-Linking

As shown in Table 3, 5,7-*O*-DA and 5,7-*O*-DM tested except for 7-*O*-A, 7-*O*-M, and 7-*O*-P demonstrated significant inhibitory activity against AGEs cross-linking. Among CS and its derivatives, 5,7-*O*-DA and 5,7-*O*-DM showed IC_50_ values of 22.33 and 39.88 μM, respectively. In contrast, 7-*O*-A, 7-*O*-M and 7-*O*-P did not inhibit AGE cross-linking and their IC_50_ values were higher than 200 μM. Similarly, 5,7-*O*-DA and 5,7-*O*-DM may be regarded as potential AGE inhibitors because of their low IC_50_ values in the three stages of AGE formation. On the other hand, CS exhibited no inhibitory activities at a concentration of 10.0 mg/mL.

### 2.6. Determination of Water Solubility

We measured the water solubility of CS and compared it with the solubility of 7-*O*-A, 5,7-*O*-DA, 7-*O*-M, 5,7-*O*-DM, and 7-*O*-P. The solubility of each compounds in water were 0.03, 0.086, 0.265, 0.010, 0.011, and 0.009 mM, respectively. The solubility of 7-*O*-A and 5,7-*O*-DA were 2.87- and 8.83-fold higher than CS.

### 2.7. Anti-Inflammatory Effect of Chrysin Derivatives on RAW 264.7 Cells

**Figure 2:**
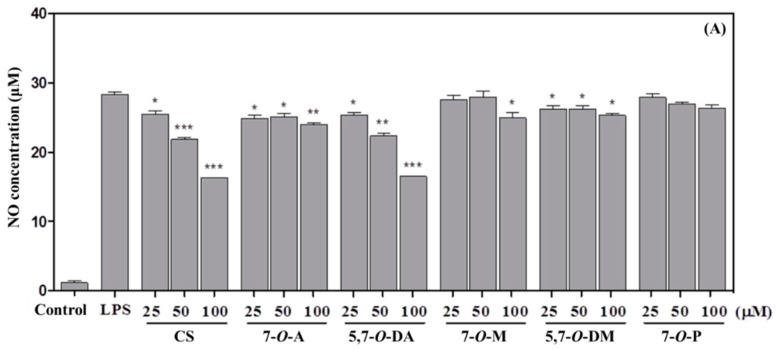

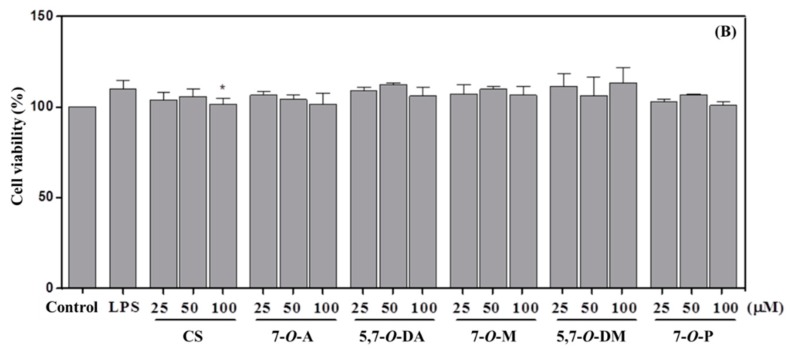
Effect of chrysin and its derivatives on NO generation (**A**) and cell viability (**B**) in RAW 264.7 cells. Asterisks indicate a significant difference compared to the LPS group (* *p* < 0.05, ** *p* < 0.01, *** *p* < 0.001). The data presented are the mean ± standard error of the mean (SEM) (*n* = 3). Chrysin (CS), 7-*O*-acetylchrysin (7-*O*-A), 5,7-di-*O*-acetylchrysin (5,7-*O*-DA), 7-*O*-methoxychrysin (7-*O*-M), 5,7-di-*O*-methoxychrysin (5,7-*O*-DM), and 7-*O*-prenylchrysin (7-*O*-P).

The effect of CS and its derivatives on LPS-induced inflammation in RAW 264.7 cells was also investigated, and NO concentration was used as a biomarker to indicate the degree of cellular inflammation. Figure 2A shows the effects of CS, 7-*O*-A, 5,7-*O*-DA, 7-*O*-M, and 5,7-*O*-DM against NO production in LPS-induced RAW 264.7 cells.

Moreover, treatment with 5,7-*O*-DA resulted in the concentration-dependent inhibition of NO in LPS-induced RAW 264.7 cells, and its effect was similar to that of CS. The cell viability effect of CS and derivatives on RAW 264.7 cells was observed by an MTS assay. The concentration of NO in the supernatant increased after LPS treatment, and no compounds exhibited cytotoxicity at concentrations between 25 to 100 μM after 24 h (Figure 2B).

## 3. Discussion

Formation of AGEs can be inhibited by interfering with the initial attachment between reducing sugars and amino groups through trapping the carbonyls and radicals formed from the glycation process, or by preventing the formation of intermediate Amadori products and blocking the formation of AGEs at the late stage of glycation [18]. Polyphenols such as stilbenes, anthocyanins, coumarins, and phenolics composed of various structures mainly scavenge MGO by trapping MGO at the active site of the aromatic ring or other positions [19,20,21,22]. Navarro & Morales (2015) reported that the α-carbon of the carbonyl group in the side chain of [6]-shogaol and [6]-gingerol is the major active site for trapping MGO [21]. Stilbenes and phenolics were detected as mono MGO adducts in the aromatic ring [20,21]. However, flavonoid MGO-conjugated adducts form by trapping MGO at the active site in the A ring of the flavonoid skeleton such as luteolin, apigenin, quercetin, kaempferol, and genistein [11,16,23,24].

**Figure 3:**
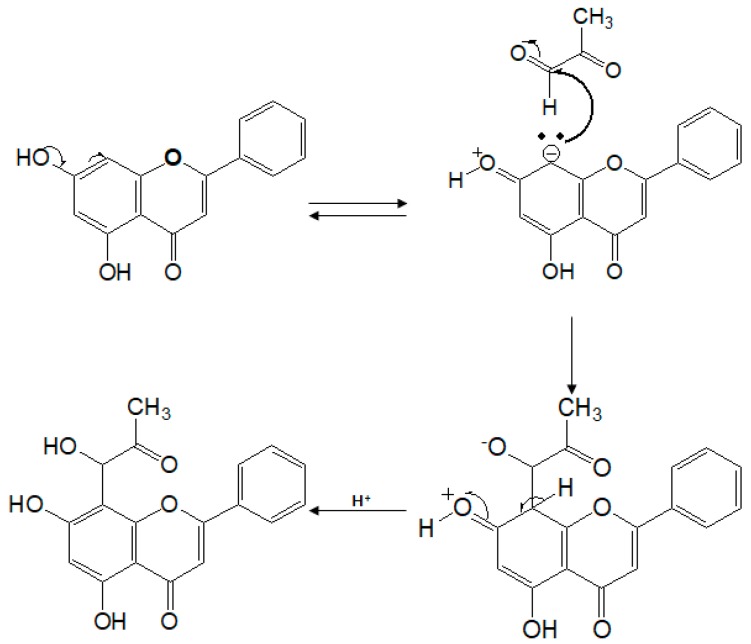
Proposed mechanism of chrysin-mono adduct formation.

It was reported that, at a slightly alkaline pH, reactive dicarbonyl intermediates such as MGO can act as nucleophilic chelaters, trappers, and conjugators [25]. Wu and Yen (2005) demonstrated that the inhibition of free radicals generation derived from glycation process was one of the mechanisms of the anti-glycation effect [26]. And Lo et al. (2011) reported the efficiency of phenols for trapping MGO [27]. Therefore, the formation of MGO-conjugated adducts might be promoted at the 6 or 6 & 8 positions as sites for nucleophilic substitutions (A ring) of flavonoids [28]. CS is reactive at the 6 and 8 positions, due to the build-up of electron density at these sites as a result of the presence of the phenol group, as illustrated by the CS-mono adduct in Figure 3.

These nucleophilic sites can attack the aldehyde group of MGO in the system to produce mono-MGO, the target molecule [22,28]. As mentioned above, we demonstrated that the intermediate formation of AGEs could be suppressed by MGO conjugation at the 6 or 6 & 8 active site of the A ring in CS. Consequently, CS in food sources may effectively trap the highly-reactive MGO by forming MGO-conjugated adducts [29].

The low solubility of flavonoids restricts their application in medicine. Therefore, many researchers have sought new methods of modifying their structures to improve their physical and physiological activities [30]. In the present study, we synthesized 5,7-*O*-DA by simple *O*-acetylation using anhydrous acetic anhydride and significantly increased the water solubility of 5,7-*O*-DA (5,7-*O*-DA > 7-*O*-A > CS > 5,7-*O*-DM > 7-*O*-M> 7-*O*-P). Furthermore, 5,7-*O*-DA efficiently inhibits AGE formation. Chen et al., (2010) reported that tetra-acetyl-luteolin exhibits increased precursor content in blood compared to that of luteolin observed upon oral administration [31]. Also, the bioavailability of acetyl-l-carnitine is 43% higher after oral administration of its precursor (l-carnitine (14–18%)) [32]. Especially, CS is quickly metabolized to CS-glucuronide by UDP-glucuronosyltransferase (Phase 2 enzyme), whereas 5,7-*O*-DA is able to increase bioavailability and can be metabolized slowly by phase 1 enzymes from human liver microsomes [33].

Many AGE inhibitors inhibit AGE formation at different stages of glycation. For example, aspirin (acetylsalicylic acid) can block the attachment between reducing sugars and amino groups by acetylating free amino groups of a protein at the early stage of the glycation process [34]. Vitamin B_1_ and B_6_ derivatives are known to scavenge reactive carbonyl compounds, and penicillamine reduces AGE yield by decreasing the formation of Amadori products [34]. When CS was synthesized to *O*-acetyl, *O*-methoxy, and *O*-prenyl structures, *O*-acetyl and *O*-methoxy derivatives exhibited stronger inhibition of AGE formation. The CS-5,7-di-*O*-acetyl, and 5,7-di-*O*-methoxy derivatives had relatively stronger activity than their 7-*O*-acetyl and 7-*O*-methoxy derivatives. The IC_50_ values of 5,7-*O*-DA for the tree steps of AGE formation were 0.26, 0.91, and 22.33 μM, respectively, while 5,7-*O*-DM appeared to have lower activity (i.e., IC_50_ values of 7.88, 41.78, and 39.88 μM) than that of 5,7-*O*-DA on the three stages. The compound 5,7-*O*-DA was more effective than CS and other derivatives at interfering with the initial attachment between reducing sugars and amino groups, trapping reactive carbonyl intermediates, and blocking the formation of AGEs. The di-*O*-acetylation at the same position elevated its inhibitory potency significantly, suggesting that the AGE inhibitory activity of CS is strongly related to the number of *O*-acetyl moieties. Similarly, 5,7-*O*-DA was more effective than CS and other derivatives at inhibiting AGE formation, and seemed to have more potential as a glycation inhibitor than AG. Additionally, flavonoids are unstable in the body since they are easily degraded by chemical and enzymatic oxidation. Therefore, it seems that *O*-acetylation increases the stability of CS by protecting it from chemical and enzymatic oxidation in cells, which may enhance its inhibitory effect on AGE production. In our study, the *O*-acetylated CS showed a similar, more effective inhibition of NO production compared to CS, which supports our hypothesis. Our data revealed that 5,7-*O*-DA had excellent AGE inhibition and high solubility, and it may serve as a potent mediator for regulating inflammation in the body. The data suggested that the di-*O*-acetylation of CS could be effective for preventing AGE formation, and therefore may help prevent and treat diabetes complications.

## 4. Materials and Methods

### 4.1. Chemicals and Reagents

Aminoguanidine hydrochloride (AG), MGO (40% aqueous solution), bovine serum albumin (essentially fatty acid-free), acetic anhydride, pyridine, magnesium sulfate (MgSO_4_), d-gluconolactone, *N*-acetyl-glycyl-lysine methyl ester acetate salt (G.K. peptide), d-ribose, d-glucose, sodium azide (NaN_3_), sodium bicarbonate, methanol (MeOH), anhydrous acetone, prenyl bromide, potassium carbonate (K_2_CO_3_), Griess reagent system, and CS were purchased from Sigma-Aldrich (St. Louis, MO, USA). The 3-(4,5-dimethylthiazol-2-yl)-5-(3-carboxymethoxyphenyl)-2-(4-sulfophenyl)-2H-tetrazolium, inner salt (MTS) assay kit was purchased from Promega (Promega Co., Madison, WI, USA). All solvents and other reagents used in this study, unless otherwise specified, were analytical grade and purchased from Sigma-Aldrich (St. Louis, MO, USA).

### 4.2. Determination of MGO Trapping Capacity by HPLC

CS (10 mM) was incubated with MGO (1, 5, 10, 50, or 100 mM) in a PBS buffer (pH 7.4, 10 mL) at 37 °C for 1, 2, 6, 12, 36, or 48 h. The incubated mixture was filtered using a Microcon YM-10 centrifugal filter unit by centrifugation at 5167× *g* for 30 min at 4 °C. The filtrate was subsequently analyzed by high-performance liquid chromatography (HPLC) using the methods mentioned in the HPLC analysis section. The samples were then stored at −80 °C for further use.

### 4.3. HPLC Analysis

HPLC was performed on an Agilent1100 series system equipped with a diode-array detector (DAD; Agilent, Sunnyvale, CA, USA) consisting of a vacuum degasser (G1322A), a quaternary pump (G1311A), an auto-sampler (G1313A), a thermostat column compartment (G1316A), and a DAD (G1315B). Separation was achieved at 30 °C on an Eclipse XDB-phenyl column (150 mm × 4.6 mm, 3.5 μm), coupled with a guard column. Sample injection volume was 10 μL. The samples were eluted with acidified water (0.1% trifluoroacetic acid, A) and MeOH (B) at a flow rate of 0.7 mL/min. The optimized gradient chromatographic conditions were 5–100% B at 0–40 min; 100–5% B at 40–42 min; and isocratic 5% B at 42–45 min. The detector monitored the eluent at a wavelength of 280 nm.

### 4.4. Isolation and Identification of Chrysin MGO-conjugated Adducts Using LC-MS/MS and NMR

MGO-conjugated adducts of chrysin were purified by using a recycle HPLC with a gradient system (0–25%, (MeOH)) as the eluent to obtain CS-mono-MGO adduct (5.14 mg) and CS-di-MGO adduct (4.83 mg). Additionally, isolated MGO-conjugated adducts of chrysin were identified as follows: (1) Liquid chromatography mass spectrometry (LC-MS/MS): The LC eluent was introduced into the ESI interface. The positive ion polarity mode was utilized for the ESI ion source. LC-MS/MS spectrum obtained using a QTRAP 4500 system (AB SCIEX, Darmstadt, Germany) with curtain gas 35 psi, ion spray voltage 5500 volts, source temperature 650 °C, nebulizer gas 55 psi, heater gas 55 psi, and scan range of 100–500 Da; (2) Nuclear magnetic resonance (NMR): Approximately 3.0–5.0 mg of each compound was dissolved in 600 μL of dimethyl sulfoxide (DMSO)-*d*6 and distributed in 3-mm NMR tubes. ^1^H and ^13^C-NMR spectra and correlation NMR spectra were obtained using an Avance DPX 400 spectrometer (Bruker, Billerica, MA, USA). Spectra were obtained at operating frequencies of 400 (^1^H) and 100 MHz (^13^C) with DMSO-*d*6, and tetramethylsilane was used as an internal standard.

### 4.5. Chrysin Derivatives Synthesis

#### 4.5.1. 7-*O*-acetyl and 5,7-di-*O*-acetylchrysin Synthesis

7-*O*-Acetyl and 5,7-di-*O*-acetylchryrin were synthesized as described previously [35]. Acetic anhydride (10 mM) was added dropwise to a solution of CS (10 mM) in 50 mL of pyridine. After 2 h of reaction at room temperature under stirring, the solvent was removed with a rotary evaporator at 40 °C. The residue was dissolved in dichloromethane (DCM), washed in 1 M HCl 3 times, then washed with saturated sodium bicarbonate solution, and water to neutralize. The organic phase was separated, dried over MgSO_4_, and concentrated in vacuo. The residue was purified by silica gel column chromatography, eluting with DCM/MeOH (10:0 to 9.5:1.5, *v*/*v*) to 7-*O*-acetylchrysin (7-*O*-A) and 5,7-di-*O*-acetylchrysin (5,7-*O*-DA).

#### 4.5.2. 7-*O*-methoxy and 5,7-di-*O*-methoxychrysin Synthesis

1,8-Diazabicyclo(5.4.0)undec-7-ene (10 mM) and CS (10 mM) were added in dimethyl carbonate (80 mL). The mixture solvent was synthesized at 100 °C for 12 h and then cooled in ice-cold water. The precipitate was separated by filtration and the solvent was removed with a rotary evaporator at 40 °C. The residue was dissolved in DCM, washed in 1 M HCl 3 times, then washed with saturated sodium bicarbonate solution and water to neutralize. The organic phase was separated, dried over MgSO_4_, and concentrated in vacuo. The resulting solid residue was purified using silica gel chromatography with DCM/MeOH (10:0 to 9.5:0.5, *v*/*v*) as the eluent, to yield 7-*O*-methoxychrysin (7-*O*-M) and 5,7-di-*O*-methoxychrysin (5,7-*O*-DM) [36].

#### 4.5.3. 7-*O*-prenylchrysin Synthesis

**Figure 4:**
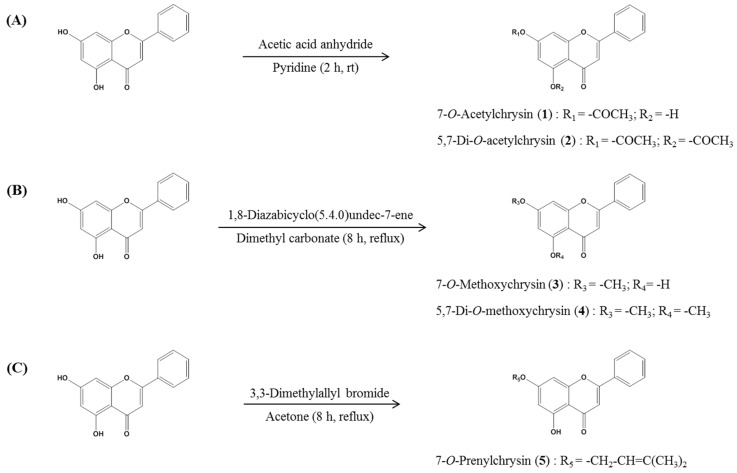
Scheme for synthesis of *O*-acetylation (**A**), *O*-methylation (**B**), and *O*-prenylation (**C**) from chrysin used in this study.

CS (10 mM), prenyl bromide (2.2 mM), and anhydrous K_2_CO_3_ (3.7 mM) in anhydrous acetone (70 mL) were refluxed at 65 °C for 8 h. The mixture solvent was removed with a rotary evaporator at 40 °C. The residue was dissolved in ethyl acetate, then purified by silica gel column chromatography, and eluted with hexane/ethyl acetate (9:1: to 2:1, *v*/*v*) to 7-*O*-prenylchrysin (7-*O*-P) [37]. The detailed NMR data and chemical structures of all CS derivatives are provided in Table S1 and Figure 4A–C.

### 4.6. AGE Formation Assay

#### 4.6.1. Hemoglobin-*δ*-gluconolactone Assay of Amadori Compound Formation

Evaluation of initial stage of PG was determined changing the method described by Hwang et al [18]. Briefly, bovine serum albumin (50 mg/mL) was incubated with glucose (144 mg/mL) in phosphate buffer (pH 7.4) containing 0.2 g/L NaN_3_ under sterile conditions in the dark at 37 °C for 48 h.

#### 4.6.2. Bovine Serum Albumin-methylglyoxal Assay on AGE Formation

Bovine serum albumin (50 mg/mL) was incubated at 37 °C for 48 h with methylglyoxal (100 mM) in sodium phosphate buffer (0.1 M, pH 7.4) containing 0.2 g/L NaN_3_ in the presence of various concentrations of the compounds (including a control) [38].

#### 4.6.3. *N*-Acetyl-glycyl-lysine-methyl Ester D-ribose Assay on AGE Cross-Linking

This test was used to evaluate the ability of samples to inhibit cross-linking of the GK peptide in the presence of d-ribose using the method described by Hwang et al [18]. The GK peptide (26.7 mg/mL) was incubated with d-ribose (200 mg/mL) in sodium phosphate buffer (0.5 M, pH 7.4) containing 0.2 g/L NaN_3_ under sterile conditions at 37 °C for 72 h. The DMSO used for dissolving samples was found to have no effect on the reaction. All reagents and samples were sterilized by filtration through 0.2 mm membrane filters. The fluorescence intensity was measured at an excitation wavelength of 355 nm and an emission wavelength of 460 nm with a Luminescence spectrometer LS50B (Perkin-Elmer Ltd., Buckinghamshire, England). AG was used as a positive control. The concentration of each test sample exhibiting 50% inhibition of activity (IC_50_) was estimated from the least squares regression line of the logarithmic concentration plotted against the remaining activity.

### 4.7. Determination of NO Generation and Cell Viability in RAW 264.7 Cells

The cytotoxicity of CS and derivatives on RAW 264.7 cells was examined using the MTS assay kit. Cells (1.6 × 10^4^/well) were cultured in 96-well plates and treated with samples (10, 25, and 100 μM) for 12, 24, 48, and 72 h. After incubation, 20 μL/well of MTS solution was incubated for 90 min at 37 °C in a humidified 5% CO_2_ atmosphere. The optical density at 490 nm was measured three times using an EL-800 Universal microplate reader (Bio-Tek Instrument Inc., Winooski, VT, USA). Cell viability of the untreated group was set to 100%. RAW 264.7 cells were seeded into 12-well plates at 4 × 10^5^-cells/well, and then incubated with LPS (1 μg/mL) and various concentrations of samples for 24 h. The concentration of nitric oxide (NO) in the medium was measured using the Griess reagent system, as described by the manufacturer. The production of NO was measured at 570 nm using an EL-800 Universal microplate reader (Bio-Tek Instrument Inc., Winooski, VT, USA), and was compared with a sodium nitrite standard calibration curve [30].

### 4.8. Solubility Analysis

CS and derivatives were dissolved in distilled water and incubated at 37 °C with sonication for 1 h to maximize solubility. After sonication, undissolved samples were eliminated by centrifugation (7000× *g*, 37 °C, 5 min). The supernatants were diluted in methanol and filtered through a 0.45 μm disposable syringe filter (Advantec, Dublin, CA, USA) to analyze the concentration of the samples using HPLC analysis [30].

## 5. Conclusions

In summary, we evaluated the AGE formation inhibitory activities of CS and generated MGO-conjugated adducts by incubating a mixture of CS and MGO. We observed the trapping of reactive carbonyls formed during glycation by LC-MS/MS and NMR spectroscopy. The results showed that CS could efficiently inhibit the initial attachment between reducing sugars and amino groups, as well as suppress reactive carbonyl compound-induced PG. In addition, among the five CS derivatives examined, 5,7-*O*-DA showed the strongest inhibition of AGE formation at three stages, and exhibited increased water solubility than CS while retaining anti-inflammatory activity. Increased water solubility and inhibitory effects of 5,7-*O*-DA may provide applicable knowledge for developing inhibitors of AGE formation, and can contribute to the development of functional food sources and beneficial materials from CS. However, physiological studies will be needed for drug development and to validate the use of these compounds as functional food sources. Moreover, research on the AGE inhibitory mechanisms of 5,7-*O*-DA is needed in the future, and it would be worthwhile to further study whether 5,7-*O*-DA can decrease the levels of reactive dicarbonyl compounds.

## Figures and Tables

**Table 1:** ^1^H & ^13^C-NMR spectra of chrysin MGO-conjugated adducts.

Carbon No.	CS		CS–Mono MGO Adduct		CS–Di MGO Adduct	
	^1^H (δ_H_)	^13^C (δ_C_)	^1^H (δ_H_)	^13^C (δ_C_)	^1^H (δ_H_)	^13^C (δ_C_)
2		163.6	-	164.2	-	164.6
3	6.3	104.6	6.29 (1H, s)	104.7	6.32 (1H, s)	104.7
4		181.3	-	180.3	-	181.6
5	12.8	161.6	-	160.1	-	162.9
6	6.8	103.3	6.83 (1H, s)	103.1	-	103.2
7	11	164.2	-	165.9	-	163.4
8	6.5	97.4	-	97.3	-	103.7
9		157.8	-	160.7	-	159.9
10		105.2	-	105.9	-	105.9
1′		130.9	-	130.9	-	130.9
2′	8	128.8	7.98–8.11 (1H, m)	128.3	7.99–8.13 (1H, m)	128.3
3′	7.6	129.6	7.68–7.59 (3H, m)	129.7	7.70–7.62 (3H, m)	129.7
4′	7.6	126.9		126.9		126.9
5′	7.6	129.6		129.7		129.7
6′	8	128.8	7.98–8.11 (1H, m)	128.3	7.99–8.13 (1H, m)	128.3
11			5.17 (1H, s)	69.9	5.18 (1H, s)	72.3
12			-	207.3	-	210.1
13			2.19 (3H, s)	25.9	2.09 (3H, s)	26.1
14			-	-	5.17 (1H, s)	72.3
15			-	-	-	210.1
16			-	-	2.09 (3H, s)	26.1

**Table 2:** HPLC area percentage (%) of chrysin-mono (A) and di-MGO (B) products upon incubation at 1–48 h at different ratios.

**Chrysin:MGO** **Incubation Ratio (A)**	**Chrysin-mono-MGO Adduct Incubation Time (h)**					
	**1**	**3**	**6**	**12**	**36**	**48**
1:10	11.9 ^a^	21.0	31.9	42.1	57.4	63.9
1:5	12.4	19.0	26.7	35.6	49.1	57.5
1:1	5.0	10.8	18.1	29.5	45.1	55.0
1:0.5	4.1	7.7	13.1	21.4	36.5	45.8
1:0.1	1.7	2.9	4.0	7.6	19.9	27.2
**Chrysin:MGO** **Incubation Ratio (B)**	**Chrysin-di-MGO Adduct Incubation Time (h)**					
	**1**	**3**	**6**	**12**	**36**	**48**
1:10	0.5	1.0	1.9	5.7	17.3	29.7
1:5	0.3	0.8	1.7	3.6	15.9	25.2
1:1	0.1	0.3	0.7	1.5	6.4	11.6
1:0.5	0.0	0.2	0.5	1.1	4.1	7.6
1:0.1	0.0	0.1	0.1	0.2	1.1	2.9

^a^ Percentage (%) = peak area of MGO-conjugated adducts produced at various incubation ratios and times by HPLC.

**Table 3:** Solubility and inhibitory effect of chrysin derivatives on three steps of advanced glycation end products (AGEs).

Compounds	IC_50_ (μM) ^a^		
	Amadori Compound Formation	AGE Formation	AGEs Cross-Linking
Chrysin	17.41	24.96	NI ^c^
7-*O*-Acetylchrysin	23.54	21.61	>2000
5,7-Di-*O*-acetylchrysin	0.26	0.91	22.33
7-*O*-Methoxychrysin	NI	>200	NI
5,7-Di-*O*-methoxychrysin	7.88	41.78	39.88
7-*O*-Prenylchrysin	NI	>200	>2000
Aminoguanidine ^b^	109.74	136.79	1902.67

^a^ The IC_50_ values are defined as the mean ± SEM of half-maximal inhibitory concentrations obtained from three independent experiments performed in duplicate. ^b^ Aminoguanidine is the positive control for AGEs. ^c^ No inhibition.

## Supplementary Materials

The following are available online. Figure S1: Solubility of chrysin derivatives. Table S1: Dose-dependent inhibition rate of CS. Table S2: ^1^H & ^13^C NMR spectra of chrysin derivatives.

## Abbreviations

AGEs	Advanced glycation end-products
MGO	Methylglyoxal
PG	Protein glycation
CS	Chrysin (5,7-dihydroxyflavone)
LC-MS/MS	Liquid chromatography mass spectrometry
NMR	Nuclear magnetic resonance
AG	Aminoguanidine hydrochloride
NaN3	Sodium azide
MeOH	Methanol
HPLC	High-performance liquid chromatography
DAD	Diode-array detector
DMSO	Dimethyl sulfoxide
DCM	Dichloromethane
7-*O*-A	7-*O*-acetylchrysin
5,7-*O*-DA	5,7-di-*O*-acetylchrysin
7-*O*-M	7-*O*-methoxychrysin
5,7-*O*-DM	5,7-di-*O*-methoxychrysin
7-*O*-P	7-*O*-prenylchrysin
G.K.peptide	*N*-acetyl-glycyl-lysine methyl ester acetate salt
