# Supplementary material for: Anti-glycation, Carbonyl Trapping and Anti-inflammatory Activities of Chrysin Derivatives
Source: Molecules. 2018 Jul 17;23(7):1752. doi: 10.3390/molecules23071752 (PMC6099615; doi:10.3390/molecules23071752)
Supplement: Supplementary file 1 [file molecules-23-01752-s001.pdf]

Table S1. ^{1}H &^{13}C NMR spectra of chrysin derivatives.

Carbon	7-*O*-Acetylchrysin		5,7-Di-*O*-acetylchrysin		7-*O*-Methoxychrysin		5,7-Di-*O*-methoxychrysin		7-*O*-Prenylchrysin	
	^{1}H (δ_H)	^{13}C (δ_C)	^{1}H (δ_H)	^{13}C (δ_C)	^{1}H (δ_H)	^{13}C (δ_C)	^{1}H (δ_H)	^{13}C (δ_C)	^{1}H (δ_H)	^{13}C (δ_C)
2	-	163.9	-	163.7	-	162.8	-	165.4	-	163.9
3	7.17 (1H, s)	104.7	6.93 (1H, s)	104.6	7.04 (1H, s)	104.9	6.78 (1H, s)	104.8	6.97 (1H, s)	104.3
4	-	182.3	-	176.3	-	181.7	-	177.6	-	181.0
5	-	160.5	-	152.3	-	161.1	-	163.1	-	161.7
6	6.69 (1H, d, 2.00)	106.4	7.64-7.57 (1H, m)	115.4	6.41 (1H, d, 2.24)	99.1	6.52 (1H, d, 2.26)	96.1	6.23 (1H, d, 2.07)	98.1
7	-	157.1	-	158.6	-	168.9	-	166.8	-	164.3
8	7.13 (1H, d, 2.00)	103.8	7.10 (1H, d, 2.34)	108.6	6.82 (1H, d, 2.32)	93.3	6.88 (1H, d, 2.21)	92.9	6.53 (1H, d, 2.08)	93.4
9	-	156.5	-	156.9	-	158.3	-	158.9	-	158.9
10	-	108.4	-	116.3	-	104.2	-	107.8	-	104.2
1′	-	130.9	-	131.4	-	132.1	-	133.1	-	130.3
2′	8.14-8.12 (1H, m)	126.3	8.10-8.07 (1H, m)	127.2	8.11-8.09 (1H, m)	128.6	8.06-8.03 (1H, m)	128.2	8.08-8.06 (1H, m)	125.9
3′		128.6		128.4		129.3		128.9		127.3
4′	7.65-7.58 (3H, m)	127.7	7.64-7.57 (3H, m)	127.3	7.63-7.57 (3H, m)	130.1	7.59-7.54 (3H, m)	130.4	7.62-7.55 (3H, m)	129.1
5′		128.6		128.3		129.3		128.9		127.3
6′	8.14-8.12 (1H, m)	126.3	8.10-8.07 (1H, m)	127.2	8.11-8.09 (1H, m)	128.6	8.06-8.03 (1H, m)	128.2	8.08-8.06 (1H, m)	125.9
5-Methoxy	-	-	-	-	-	-	3.90 (3H, s)	56.3	-	-
7-Methoxy	-	-	-	-	3.88 (3H, s)	56.1	3.84 (3H, s)	56.3	-	-
5-Acetyl	-	-	-	169.5	-	-	-	-	-	-
5-Acetyl-CH_3	-	-	2.17 (3H, s)	21.1	-	-	-	-	-	-

7-Acetyl	-	169.4	-	169.5	-	-	-	-	-	-
7-Acetyl-CH_3	2.32 (3H, s)	21.4	2.17 (3H, s)	21.3	-	-	-	-	-	-
7-Prenyl-1′	-	-	-	-	-	-	-	-	4.58 (2H, d, 6.60)	65.0
7-Prenyl-2′	-	-	-	-	-	-	-	-	5.49 (1H, br, s)	118.7
7-Prenyl-3′	-	-	-	-	-	-	-	-	-	138.2
7-Prenyl-CH_3	-	-	-	-	-	-	-	-	1.77, 1.82 (6H, s)	18.3, 25.2
